# Targeting the synovial engine: next-generation engineered immune cells to eradicate pathogenic FLS in rheumatoid arthritis, with safety-first, selective designs

**DOI:** 10.3389/fimmu.2026.1890594

**Published:** 2026-07-09

**Authors:** Ashik Anil Mathew, Arulkumaran Rithvik, Mahaboobkhan Rasool

**Affiliations:** Immunopathology Lab, School of Biosciences and Technology, Vellore Institute of Technology (VIT), Vellore, Tamil Nadu, India

**Keywords:** chimeric antigen receptor, fibroblast-like synoviocytes, rheumatoid arthritis, safety engineering, synovium, targeted cell therapy

## Abstract

Rheumatoid arthritis (RA) and associated inflammatory arthritides are characterized by chronic synovial inflammation that drives destructive pannus formation, eroding cartilage and bone. Current therapies (csDMARDs, bDMARDs, and tsDMARDs) target immune cells and cytokine pathways but yield low rates of full remission, and many patients with residual synovitis continue to damage their joints. Fibroblast-like synoviocytes (FLS) have emerged as key tumor-like mediators of RA. In the inflamed synovium, they expand, resist apoptosis, and secrete pro-inflammatory cytokines, chemokines, and proteases that sustain disease. Directly killing and/or reprogramming these pathogenic FLS is a promising complementary strategy. Recent advances in engineered cell therapies enable specific targeting of stromal cells. In this review, we will discuss novel CAR-based immune cells (CAR-T, CAR-NK, or CAR-macrophages) engineered to target FLS-specific antigens (e.g., fibroblast activation protein) and induce elimination or modulation of FLS. We advocate a primary “safety-first” translational roadmap that integrates features such as transient CAR expression, safety switches, hypoxia-responsive CAR constructs, and localized (intra-articular) delivery to enhance efficacy while avoiding systemic toxicity. Targeted cellular therapies based on this technology may reshape RA therapy as we know it, dismantling the synovial inflammation “engine” with precision and control.

## 1 Introduction

Rheumatoid arthritis is a common autoimmune disorder affecting almost 0.5–1% of the population. RA is marked by chronic synovial inflammation, pannus hyperplasia, and progressive destruction of cartilage and bone (1). Although conventional and biologic disease-modifying antirheumatic drugs (DMARDs), including anti-TNF and anti-IL-6 antibodies and JAK inhibitors, have substantially improved clinical outcomes, durable drug-free remission remains uncommon, and a substantial subset of patients show inadequate response, secondary loss of response, or intolerance to targeted therapy. In particular, up to approximately 40% of patients may respond inadequately to an initial anti-TNF agent, and real-world analyses show that a clinically relevant proportion of RA patients discontinue, switch, or cycle through biologic/targeted synthetic DMARDs because of inefficacy, loss of response, or adverse events (2, 3). Recent work has placed stromal cells, particularly fibroblast-like synoviocytes, at the center of RA pathogenesis. FLSs are the dominant resident cell population in inflamed synovium and adopt an aggressive phenotype in RA, showing hyperproliferation, resistance to apoptosis, and excessive secretion of pro-inflammatory cytokines (for example, IL-6 and type I interferons), chemokines, and matrix metalloproteinases (4). Single-cell and spatial transcriptomic profiling have identified discrete pathogenic FLS subsets with distinct gene-expression signatures, and these “fibroblast signatures” are associated with treatment failure (5, 6). Together, these findings argue that pathogenic FLS are active architects of persistent synovial inflammation and highlight them as compelling targets for new therapeutic strategies. Existing therapies largely ignore stromal drivers, and directly targeting pathogenic FLS could overcome some limitations of conventional treatments. For example, synovial biopsies from RA patients who failed biologics (e.g., anti-IL-6 or anti-CD20) show a relative enrichment of fibroblast-related gene expressions. In such a fibroblast-rich synovial environment, targeting immune depletion is ineffective. Whereas targeting and eliminating pathogenic fibroblasts might prevent disease progression and have been tested successfully in preclinical trials, none have benefited in clinical settings (7). This underscores the need for stronger target validation, careful patient selection, and safety-focused therapeutic designs before these approaches can be advanced into clinical practice.

Cell-based immunotherapies have transformed medicine: the adoptive transfer of genetically engineered cells, perhaps most prominently CAR-T, has shown that programmable “living drugs” yield deep, durable remissions, and this clinical success ignited efforts to develop CAR and related platforms towards non-malignant disease (8). More recently, the concept of engineered immune cells has expanded into the realm of autoimmune diseases (9). A case series study was conducted in 15 patients with refractory SLE, idiopathic inflammatory myopathy, and systemic sclerosis who showed clinical benefit from CD19 CAR-T cell immunotherapy, with a median follow-up of 15 months. In this study, clinical benefits were observed in all patients with cessation of the immunosuppressive therapy regimen, while adverse effects included grade 1 CRS in 10 patients, grade 2 CRS in 1 patient, grade 1 ICANS in 1 patient, and pneumonia in 1 patient. A 2026 case report described treatment-free remission for 11 months in a patient with refractory autoimmune hemolytic anemia (AIHA) with concomitant ITP and antiphospholipid syndrome following CD19 CAR-T cell therapy. Nonetheless, these findings have only been reported in single-case studies and case series, underscoring the need for longer follow-up and controlled clinical studies (10, 11). Extending this paradigm to rheumatoid arthritis, CAR-engineered effectors, including T cells, NK cells, and macrophages, can be directed to synovial stromal antigens such as fibroblast activation protein and other RA-enriched markers. This will selectively ablate or reprogram pathogenic fibroblast-like synoviocytes. with preclinical and transient *in vivo* CAR generation approaches demonstrating fibroblast depletion and tissue remodeling (12). Importantly, translation to a non-oncologic setting requires a “safety-by-design” mindset: affinity-tuned and combinatorial antigen recognition (synNotch/AND-gates); transient or nonintegrative CAR expression (mRNA/LNP or episomal platforms); rapid pharmacologic off-ramps (inducible suicide switches); effector selection for limited persistence and attenuated cytokine profiles (CAR-NK, CAR-M); and localized intra-articular delivery or retention strategies (13, 14). In this review, we evaluate next-generation engineering solutions, modular CAR architectures, controllable expression systems, effector-cell choice and delivery paradigms, and outline a pragmatic translational roadmap to achieve durable, drug-free remission in RA while minimizing systemic risk.

## 2 The synovial engine: FLS biology and its pathogenic states

**Figure 1:**
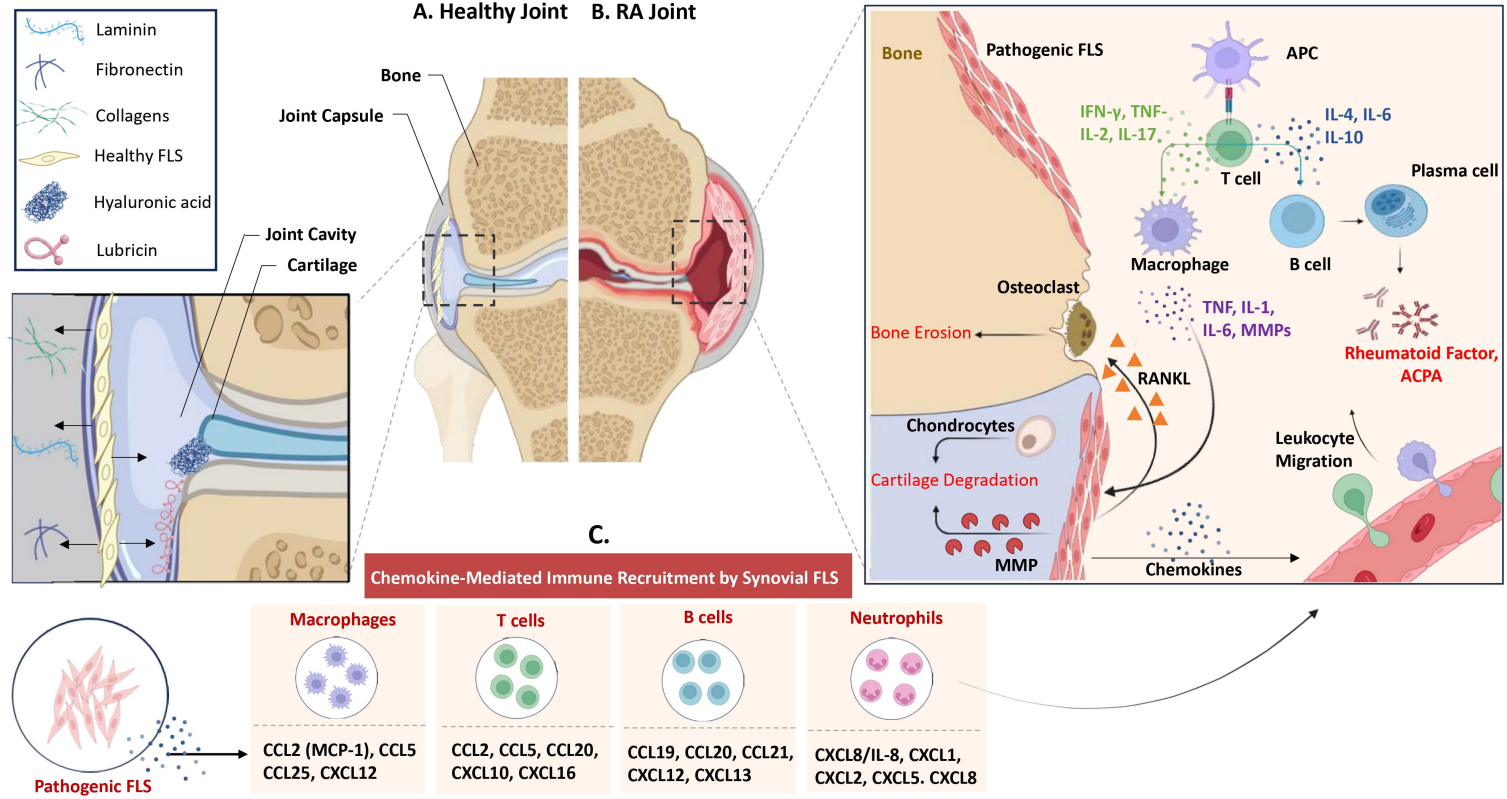
Healthy versus rheumatoid arthritis synovial microenvironment and the central role of fibroblast-like synoviocytes in joint destruction. **(A)** In healthy joints, the synovial lining preserves tissue homeostasis by maintaining a well-organized extracellular matrix composed of laminin, fibronectin, collagens, hyaluronic acid, and lubricin. **(B)** In rheumatoid arthritis, synovium undergoes hyperplasia and becomes highly inflamed, leading to pannus formation, cartilage destruction, and bone erosion. Pathogenic FLS further amplifies inflammatory responses and joint damage. Pathogenic FLS also release chemokines that attract macrophages, T cells, B cells, and neutrophils into the joint, thereby sustaining synovial inflammation. **(C)** Pathogenic FLS release chemokines that recruit macrophages, T cells, B cells, and neutrophils into the inflamed joint, thereby sustaining synovial inflammation and contributing to cartilage destruction and bone erosion.

The synovium is a thin, delicate membrane normally consisting of an intimal layer covered with fibroblast-like synoviocytes (FLS; type B) and macrophage-like synoviocytes (type A) over sparsely cellular sublining, whose main tasks in homeostasis are secretion of hyaluronan and lubricin (PRG4) by FLS, as well as debris clearance and innate defense through activity of the type A cells (15). This brings us to the synopsis of RA pathophysiology, where this architecture is completely remodeled: both lining and sublining are characterized by intimal hyperplasia, with a normally 1–2-cell-deep lining that can expand to 10–20 cell layers; the capsule becomes a highly vascularized site of heavy macrophage, lymphocyte, and other leukocyte infiltration (16). In early disease, there is angiogenesis and leukocyte influx, followed by the formation of organized lymphoid aggregates and an invasive pannus that causes erosion of cartilage and bone. And so, the RA synovium “evolves from a quiescent, relatively acellular structure to a hyperplastic, invasive tissue saturated with immunocompetent cells” (17). Fibroblast-like synoviocytes (FLS, type B synoviocytes) constitute this intimal lining and are defined by expression of mesenchymal markers, e.g., vimentin and CD90/Thy-1, along with basement-membrane collagens (types IV/V) and the adhesive molecule cadherin-11; physiologically they secrete hyaluronan and lubricin (with UDP-glucose dehydrogenase providing HA precursors) to maintain joint lubrication but in RA undergo dramatic hyperplasia, acquire an aggressive invasive phenotype that proliferates, migrate into cartilage and drive matrix destruction. Sitting within the lining are bone-marrow-derived macrophage-like synoviocytes (type A; CD11b+, CD68+) that expand and activate in RA and secrete TNF-α, IL-1, IL-6, and chemokines that further drive inflammation and reciprocally activate FLS (18–20) (**Figure 1**).

The sublining microvasculature undergoes robust angiogenesis in response to synovial hypoxia, thereby increasing perfusion and leukocyte recruitment into the inflamed synovium. The cellular infiltrate is dominated by adaptive immune cells, particularly CD4^+^ memory T cells, which account for approximately 30–50% of infiltrating cells, along with smaller populations of CD8^+^ T cells, B cells, plasma cells, dendritic cells, mast cells, and monocytes. These immune cells may appear as diffuse infiltrates or organize into lymphoid-like aggregates, where B cells and plasma cells can support local autoantibody production (21, 22). Osteoclast precursors attracted to the pannus interface differentiate by RANKL signals and drive focal bone resorption; neutrophils, in contrast, are primarily localized to synovial fluid rather than tissue (23, 24). Together, these establish a self-perpetuating paracrine network in which macrophage TNF-α/IL-1 and chemokines drive FLS production of IL-6, prostaglandins, and matrix metalloproteinases, which further attract effector cells while promoting cartilage erosion, generating the defining RA histopathology comprising thickened lining, inflamed sublining, and invasive pannus (25). Recent single-cell and spatial transcriptomic studies, together with functional and imaging data, have refined our understanding of pathogenic FLS and macrophage subsets in RA synovium. By identifying niche-specific metabolic and hypoxia-driven programs, these advances now support more precise, tissue-directed strategies to deplete or reprogram destructive synovial cell populations (26, 27).

### 2.1 FLS heterogeneity and pathogenic phenotypes

**Figure 2:**
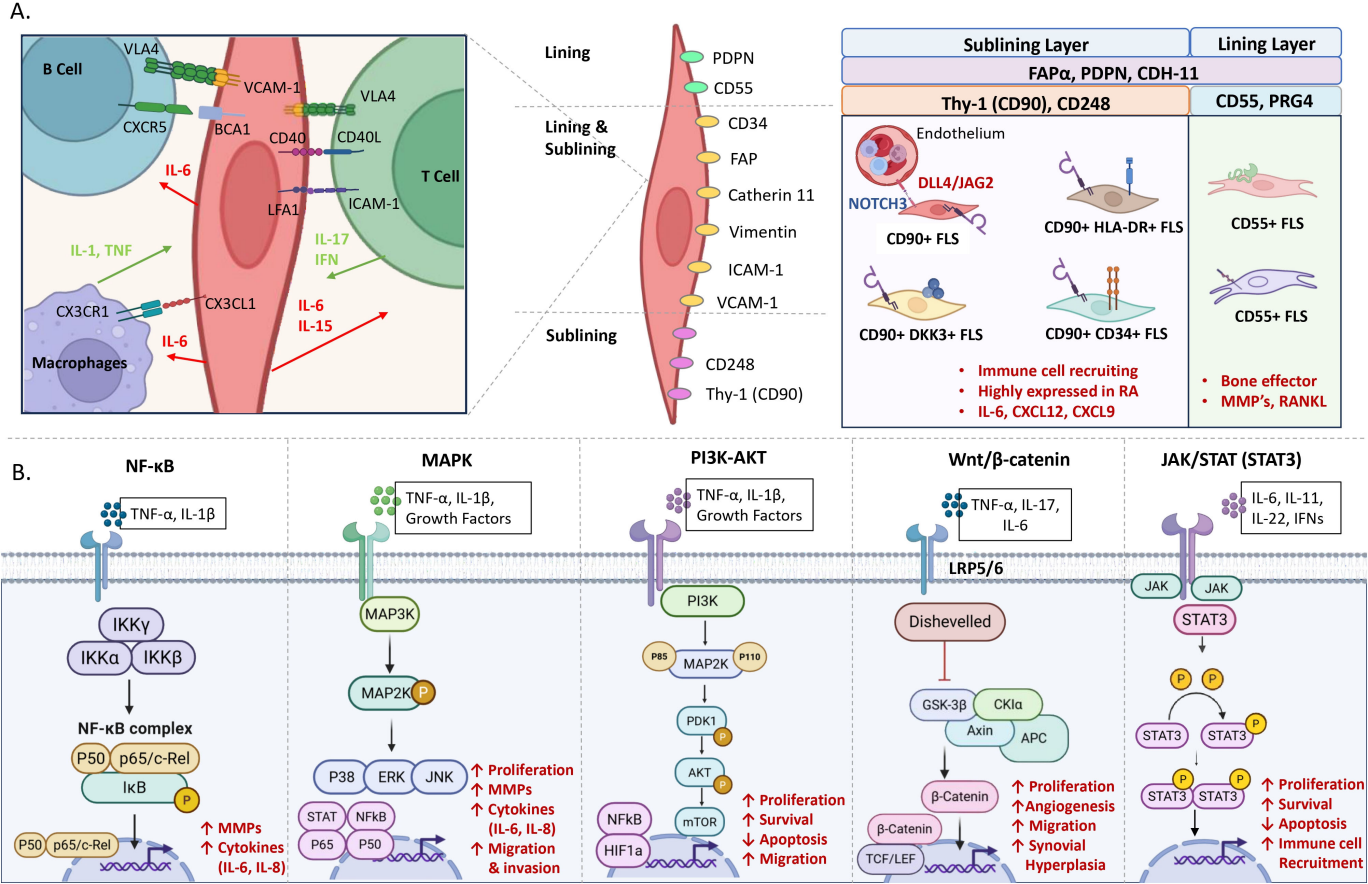
Heterogeneity of rheumatoid arthritis fibroblast-like synoviocytes and the signaling pathways driving their pathogenic activation. **(A)** Distinct FLS populations reside in the lining and sublining layers of the synovium, each defined by specific markers. These cells communicate with immune cells, including T cells, B cells, macrophages, and endothelial cells, thereby driving synovial inflammation and joint damage. **(B)** In rheumatoid arthritis, FLS pathogenicity is largely governed by the NF-κB, MAPK, PI3K–AKT, Wnt/β-catenin, and JAK/STAT3 pathways, which promote FLS activation, proliferation, migration, cytokine production, and matrix degradation.

High-dimensional single-cell and spatial transcriptomic studies challenged the concept of a homogeneous synovial fibroblast and defined distinct, spatially organized FLS programs in RA (28). Single-cell RNA sequencing divides lining-layer versus sublining FLS states: active lining clusters in inflamed tissue upregulate inflammatory and matrix-remodeling genes (such as FN1, MMP3, and HLA-DR), while a “resting” lining program enriched in remission expresses lubricating and ECM genes (PRG4, CLIC5, and CD55) (29). A prominent THY1+ (CD90+) sublining fibroblast population, partially induced by NOTCH3 signaling in response to endothelial Jagged/DLL4 ligands, represents a robust source of IL-6 and other pro-inflammatory mediators (30, 31). These niche signals, along with spatial cytokine cues (TNF-α, IL-1β, and IFN-γ), drive at least four transcriptionally and functionally distinct FLS states in RA. Despite this heterogeneity, pathogenic RA-FLS coalesce on a shared effector program not present at healthy synovium: cell–cell and cell–matrix adhesion (most notably cadherin-11 with high expression of VCAM-1, ICAM-1 and α4β1 integrin); the prolific secretion of proteases (MMP−1/-3/-9/-13 and ADAMTS−4/5) that cleave collagen and proteoglycans; a pro-inflammatory secretome (IL-6, IL-8, GM-CSF and IL-15 plus chemokines like CCL2/CXCL12). Such a phenotype is exemplified by HLA-DR+ FLS, with co-expression of IL-6, IL-15, CCL2, CXCL9/CXCL12, and adhesion molecules coupled to the downregulation of homeostatic/lubricating genes, providing a molecular switch that mediates the FLS “aggressive” phenotype that fuels pannus formation and joint destruction (32–34) (**Figure 2A**).

### 2.2 Functional contributions of pathogenic FLS

Pathogenic fibroblast-like synoviocytes are central executors of joint destruction in rheumatoid arthritis. By acting as a locally invasive tissue, the pannus expands over articular cartilage. It secretes prodigious quantities of proteases, matrix metalloproteinases, and ADAMTS aggrecanases that cleave collagen and aggrecan fibrils, expressed at levels well beyond those of normal fibroblasts (35). Invasive FLS are the primary cells degrading cartilage, a conclusion corroborated by cadherin-11 knockout models (which do not generate a proper synovial lining) in which much of the cartilage is preserved (36). Recent work by Laragione et al. further strengthens this concept by identifying the transcription factor DLX4 as a key regulator of RA-FLS invasiveness, linking these cells to a distinct cancer-like transcriptomic signature and underscoring striking similarities between aggressive synoviocytes and metastatic cells (37). Simultaneously, FLS perpetuate bone destruction by orchestrating osteoclastogenesis by the production of RANKL and M-CSF (among other osteoclastogenic mediators) so that inflammatory FLS together with osteoclasts, populate the destructive pannus (38). Recent work by Lee et al. (2025) describes a feed-forward interaction in which calcium and cytokines released by osteoclasts further activate FLS and enhance their tissue-destructive enzyme program. Thus, while osteoclasts directly mediate mineralized bone resorption, FLS help initiate and amplify this destructive process by organizing the pannus and sustaining catabolic gradients that drive joint damage (39). In addition to tissue degradation, FLS are highly involved in immune crosstalk. They are not passive bystanders but respond to and shape inflammation. Pro-inflammatory cytokines from T cells (e.g., IFN-γ, TNF-α) upregulate FLS chemokine production, creating chemotactic gradients that recruit more immune cells. Pathogenic RA-FLS acquire their aggressive phenotype through sustained activation of multiple intracellular signaling networks. Among these, NF-κB, MAPK, PI3K/AKT/mTOR, Wnt/β-catenin, and JAK/STAT are the principal pathways that promote inflammation, survival, invasion, and matrix degradation (40–42) (**Figure 2B**).

At the same time, activated FLS themselves express MHC class II and costimulatory molecules and can present antigens to CD4+ T cells *in vitro*. As discussed above, HLA-DR+ RA-FLS secrete IL-6, IL-15, and several chemokines (CCL2, CXCL9/CXCL12) that recruit monocytes and T cells. In contrast, TNF-α/IL-1 from macrophages serves to enhance FLS activation (43, 44). This paracrine feedback loop (macrophage • FLS • cytokines/chemokines • leukocytes) promotes synovitis. In summary, FLS are bare targets and sustainers of immune response in joints (45). Notably, pathogenic FLS are resurrected and resistant to the homeostatic regulation seen in the majority of normal fibroblasts: chronic NF-κB signaling and aberrant regulation of p53 pathways in inflamed synovium render them refractory to apoptotic signals, enabling fibroblasts to persist despite DNA damage while retaining a hyperplastic, destructive state that not only destroys cartilage but also promotes osteoclast-mediated bone erosion (46, 47). In addition to survival, new data suggest that FLS can acquire a form of inflammatory “memory” (e.g., through metabolic and possibly epigenetic reprogramming); mouse models show how repetitive inflammatory insults prime synovial fibroblasts to become hyper-responsive with increased migratory and invasive behavior upon challenge, and although the degree of analogous imprinting in human RA is still being actively investigated, this persistence as well as priming can explain chronicity and flare relapse (48–50). Collectively, these properties, immune crosstalk, antigen-presenting capacity, pro-inflammatory secretome, apoptosis resistance, and trained hyper-responsiveness, delineate a pathogenic FLS phenotype that acts as an engine of joint destruction and a central reservoir perpetuating chronic rheumatoid synovitis.

## 3 Target discovery and antigen selection for FLS−directed therapy

### 3.1 Principles of ideal target selection

Given the transcriptional and functional heterogeneity of FLS subsets in RA synovium, a therapeutically effective antigen should ideally exhibit high disease specificity with minimal expression in healthy tissues. At a practical level, this requires identifying a cell-surface protein that is highly and uniformly expressed on pathogenic FLS clones that mediate synovial inflammation, but is essentially absent or present at very low levels on quiescent FLS in non-diseased synovium and on fibroblasts in vital organs (51). Cell-surface localization with an exposed extracellular domain is a prerequisite for antibody binding or recognition by CAR-engineered immune cells (52). Furthermore, receptor trafficking dynamics may elucidate the optimal therapeutic modality for targeting: rapidly internalizing targets are more amenable to antibody–drug conjugates, whereas stably expressed and persistently displayed surface molecules are better suited to CAR-based approaches (53). Cadherin-11 (CDH11) is a very promising example, showing enriched expression in the rheumatoid arthritis synovium but comparatively low levels in most normal, non-inflamed fibroblasts (54). In contrast, widely expressed fibroblast markers such as vimentin or markers shared with important non-FLS lineages are more likely to result in on-target, off-tissue toxicity. As such, pinpointing effective therapeutic targets must integrate single-cell and spatial expression atlases with protein-level validation and functional assays to assess both efficacy and safety in a therapeutic context (55).

Recent single-cell RNA sequencing studies have demonstrated that the RA synovium harbors multiple transcriptionally and functionally distinct FLS populations, comprising lining and sublining cells, as well as inflammatory and bone-effector subsets (56, 57). Thus, for an immunotherapy target to be effective, it needs to be broadly distributed among the pathogenic FLS populations that drive disease rather than confined to a single niche. In practice, this would translate to using a cell-surface antigen with robust expression in most pro-inflammatory FLS, e.g., the common lining- and sublining-compartment resident markers FAPα or PDPN.CD90/THY1 illustrates this complexity: within RA synovium, it is useful for identifying inflammatory sublining fibroblasts, but it does not represent all FLS subsets associated with RA pathology. It is expressed in various non-synovial tissues and other cell types besides FLSs. Thus, CD90/THY1 would be more appropriate as an FLS subset marker, for patient stratification, or as a component of a combinatorial approach rather than a single-target CAR. In light of the lack of a pan-FLS marker specific to RA, a combinatorial approach is necessary (58). No less crucial is reproducibility among patients and across species for translation: there should be a high prevalence of candidate antigens in cohorts with human RA (not rare, patient-specific variants) and ideally conservation in the preclinical species chosen for efficacy and toxicology studies (for example, genetic loss-of-function mice have lent support to the relevance of human targets exemplified by CDH11 and α11-integrin), or humanized systems must be used (59). Further, off-target expression and receptor trafficking have a significant impact on modality choice and safety profiling: targets expressed on key non-FLS lineages (such as PDPN in lung alveolar epithelium and lymphatic endothelium or THY1 detectable on neuronal and thymic cells) can predispose to collateral injury, whilst relatively low-level expression confined to non-critical sites is permissible provided there is a therapeutic window (as early FAP-targeting CAR-T work in oncology suggests) (60, 61). Taken together, only a limited set of antigens presently meets the combined criteria of disease specificity, functional relevance, and translational promise.

### 3.2 Candidate surface targets

#### 3.2.1 FAP (Fibroblast activation protein α)

Fibroblast activation protein-α (FAPα) is a type II transmembrane serine protease that is robustly upregulated on activated fibroblast-like synoviocytes FLS in RA and has emerged as a leading immunotherapeutic candidate (62). Multiple groups report dense FAP immunostaining concentrated in the synovial lining of inflamed RA joints. At the same time, healthy synovium and osteoarthritic tissue show little to no signal (Bauer et al., 2006 described high-density FAP co-localizing with matrix metalloproteinases in intensely inflamed RA samples). Contemporary reviews continue to classify FAP as a canonical activation marker with low baseline expression in most normal tissues (63). Clinically, 68Ga–FAPI PET imaging vividly localizes inflamed synovia in RA patients, providing *in vivo* confirmation of elevated FAP (64, 65). FAP’s cell-surface localization and enrichment on myofibroblast-like pathological stromal cells make it amenable to both antibody-based and cell-based modalities, and functional studies show that depletion of FAP^+^ cells in murine arthritis models markedly reduces synovitis and bone erosion (51, 66). Translational momentum is further supported by FAP-directed imaging, vaccination, photodynamic therapy, and cell-therapy approaches across inflammatory and fibrotic disease settings; however, these data should be interpreted as proof of concept rather than definitive safety validation for systemic FAP depletion in RA (67). Nevertheless, important safety caveats remain: FAP is also induced in tumor stroma, healing wounds, and fibrotic lesions, and genetic or pharmacologic FAP ablation in mice has been associated with cachexia and anemia in some settings, indicating potential on-target, off-tissue liabilities (68). In mouse inflammatory arthritis models, FAP deficiency or depletion of FAPα+ fibroblasts reduced the severity of inflammatory arthritis, synovitis, cartilage damage, and/or bone erosion. At the same time, an FAP mRNA-LNP vaccine suppressed disease in murine CIA/CAIA models. In contrast, FAP-targeted photodynamic approaches in RA are currently supported mainly by human imaging and ex vivo synovial explant data rather than *in vivo* RA efficacy studies (51, 67, 69). Collectively, the weight of pathological, imaging, and interventional data positions FAP as a compelling but not risk-free target for RA FLS–directed therapies.

#### 3.2.2 Cadherin-11 (CDH11)

Cadherin-11 (CDH11) is a cell–cell adhesion molecule that mediates fibroblast contacts and exhibits a synovium-centric expression pattern, positioning it as a leading candidate for FLS-directed therapy in rheumatoid arthritis (70). CDH11 is abundant in synovial FLS, especially lining fibroblasts, by immunohistochemistry and transcriptomics during normal homeostasis; very low-level expression is found in other quiescent connective tissues, while global deficiency leads to a thin, hypoplastic synovial lining and resistance to experimental arthritis, indicating both its role in form and pathological importance (71). Functionally, CDH11-positive FLS are proinflammatory secretors of cytokines (e.g., IL-6) and other mediators associated with joint injury. The frequency of circulating CDH11-expressing precursors increases before clinical flares, which are associated with CDH11 and disease activity (72). Advantages in targeting include relative fibroblast specificity compared with pan-stromal markers, proof of concept for genetic and blocking studies that reduce synovitis and bone erosion, and early clinical testing, with a humanized anti-CDH11 antibody, RG6125, entering a phase 2 RA study (73). Off-target liabilities seem limited but not absent: low-level expression in lung and bone marrow mesenchyme means systemic blockade must be approached with caution and appropriate safety monitoring. However, no major clinical safety signals have been observed to date (74, 75). In aggregate, the pathological enrichment, mechanistic contribution to the pathophysiology of synovitis, preclinical mitigation of disease in genetic and antibody inhibition models, and initial exposure in human clinical studies make CDH11 a leading, translationally validated FLS antigen; it warrants protein-level mapping and safety testing before more widespread therapeutic consideration (76).

#### 3.2.3 Podoplanin (PDPN/gp38)

Podoplanin (PDPN, also known as gp38) is a mucin-type transmembrane glycoprotein and a well-recognized marker of activated, invasive fibroblast-like synoviocytes in RA. Immunohistochemical studies show strong PDPN staining localized to the hyperplastic synovial lining of inflamed joints, with little to no expression in healthy or osteoarthritic synovium, and single-cell and spatial transcriptomic analyses have confirmed expansion of PDPN^+^ FLS populations in RA tissues (77). In terms of function, PDPN-expressing FLS show aggressive characteristics in preclinical models, with a migration-competent phenotype capable of invading cartilage in xenograft and mouse assays and stimulating osteoclastogenesis, consistent with a role in tissue destruction (59). Importantly, the best-characterized PRIME-cell study identified these flare-associated cells as circulating CD45−CD31−PDPN+ pre-inflammatory mesenchymal cells that expand in blood approximately 1–2 weeks before RA flares and share transcriptional features with inflammatory synovial fibroblasts, supporting PDPN as a flare-associated mesenchymal marker rather than defining PRIME cells as CDH11-positive precursors (78). These attributes render PDPN an appealing candidate for certain targeted strategies: its localization in the cell surface and upregulation restricted to diseased states favor antibody- or cell-based modalities, and oncology groups have already advanced PDPN-directed CAR-T and antibody programs, demonstrating translational tractability (79). However, off-tissue expression limits systemic targeting; PDPN is physiologically expressed in the lymphatic endothelium as well as on renal podocytes and type I alveolar epithelial cells, so systemic blockade risks lymphatic, renal, or pulmonary effects (60). Thus, RA strategies should judiciously consider modality and delivery (e.g., localized cell therapy, bispecifics, or dosing regimens that spare critical compartments) and depend on rigorous protein-level mapping and virtual safety profiling before clinical translation.

#### 3.2.4 Thy-1 (CD90)

Thy-1 (CD90) is a glycosylphosphatidylinositol-linked surface glycoprotein that defines a specific pathogenic group of sublining fibroblast-like synoviocytes in RA. CD90^+^ FLS are abundant in the inflamed sublining layer and often express activation-associated markers such as FAP and PDPN. Functionally, the CD90^+^ sublining FLS exhibit a pro-inflammatory phenotype, characterized by enhanced expression of cytokines such as IL-6 and CXCL12 (31, 80). In turn, CD90^-^ lining fibroblasts are mostly associated with osteoclastic differentiation and bone remodeling. This functional separation was demonstrated using mass cytometry and single-cell approaches to study the RA synovium, revealing a large population of THY1^+^HLA-DR^hi fibroblasts associated with the recruitment of inflammatory leukocytes and pathological inflammation. Finally, according to preclinical model results, CD90^+^ stromal cells appear to play a greater role in the development of synovitis than CD90^-^ fibroblasts (81, 82). These findings position CD90 as an excellent biomarker for inflammatory sublining FLSs, but not as a single-antigen targeting therapy. This is critical for designing CAR constructs. A single-antigen approach using a CD90 CAR will selectively eliminate CD90+ inflammatory stromal cells; however, the CD90−FAP+/PDPN+ lining FLS will be missed and remain capable of stimulating osteoclast function, cartilage invasion, and bone degradation. Thus, while CD90 is useful for defining the inflammatory fibroblast microenvironment, it does not capture the full range of disease-causing RA-FLS. Here, CD90 is seen less as an independent therapeutic target and more as a contextual biomarker (28, 51).

Safety considerations further limit the use of CD90 as a single depletion antigen. CD90 is not restricted to RA synovium and is also expressed by several physiological cell populations, including mesenchymal stromal cells, endothelial cells, neurons, thymocytes, and progenitor cells (83, 84). Direct systemic depletion of CD90-positive cells could therefore create substantial on-target, off-tissue toxicity. A more rational strategy would be to incorporate CD90 into multi-antigen or logic-gated CAR systems. For example, a CD90-sensing synNotch receptor could be used to induce a CAR directed against a second RA-enriched stromal antigen, such as FAP, PDPN, or CDH11, thereby restricting effector activity to CD90-rich inflammatory synovial niches. Conversely, in erosive RA, where CD90^-^FAP^+^PDPN^+^ lining fibroblasts may drive structural damage, CD90 should not be used as an obligatory AND-gate. Instead, dual-target, OR-gate, or modular CAR designs may be required to cover both CD90^+^ inflammatory sublining fibroblasts and CD90^-^ destructive lining fibroblast states. Thus, the biology of CD90 strongly supports multi-targeted and context-dependent CAR design rather than single-antigen FLS depletion (85–87).

#### 3.2.5 uPAR (CD87) and integrins

Urokinase plasminogen activator receptor (uPAR, CD87) is often overexpressed on RA FLS. It acts as a multifaceted mediator of matrix remodeling, cell migration, and pro-angiogenic signaling through integrin interactions and downstream kinases (88). This biology makes the uPAR–integrin axis an appealing pathway-level target to restrict FLS invasion and neovascularization. However, the broad expression of uPAR on many circulating myeloid cells and endothelium, as well as proteolytic shedding that yields soluble receptor species, makes direct targeting difficult and poses the risk of disrupting normal host defense and wound repair mechanisms. Therefore, pathway modulation or context-restricted delivery may be a preferable route to indiscriminate depletion (89). Among integrins, collagen-binding α11β1 has been identified as a fibroblast-enriched receptor associated with adhesive and tissue-destructive programs in inflamed synovium, and its relatively restricted stromal distribution, compared with more common integrins, makes it a particularly interesting antigen candidate for FLS-directed therapies (90). On the other hand, various integrins are widely distributed across tissues and primarily mediate general adhesive functions, thereby limiting their specificity for RA.

#### 3.2.6 CXCL12 (SDF-1)/CXCR4 axis

The CXCL12/SDF-1–CXCR4 axis is a relevant chemokine pathway in the RA synovium but cannot be considered equivalent to classical cell-surface targets of RA-FLS, such as FAP, PDPN, or CDH11. RA-FLS is capable of secreting CXCL12 and, upon stimulation by inflammatory or hypoxic conditions, expresses CXCR4, allowing autocrine and paracrine signaling pathways responsible for causing fibroblast migration, leukocyte recruitment, leukocyte retention, angiogenesis, osteoclastogenesis, cross-talk, and tissue invasion locally (91). The experiments carried out on collagen-induced arthritis have shown the pathogenic importance of the ligand-receptor complex by showing that the use of pharmacologic CXCR4 inhibitor (AMD3100) prevented arthritis development, restricted CXCL12-induced leukocyte recruitment, and prevented CXCL12-induced osteoclast differentiation and bone resorption (92). Nevertheless, despite high disease relevance, CXCR4 is not appropriate for CAR-based depletion. CXCR4 is widely expressed on lymphocytes and hematopoietic stem/progenitors, whereas CXCL12 is an important factor in regulating the bone marrow microenvironment and the retention of hematopoietic cells. A clinical example of this is AMD3100, which mobilizes hematopoietic stem cells into circulation. Hence, irreversible depletion of CXCR4^+^ cells is not feasible in a chronic non-cancerous condition such as rheumatoid arthritis (93, 94).

Rather, a more pragmatic approach would be to focus on the use of CXCL12/CXCR4 as a pharmacologically targetable stromal-immune retention axis instead of a single-target CAR therapy. Under this construct, CARs targeting the stroma (FLS) in terms of molecules that are enriched in the synovium (FAP, PDPN, CDH11, and integrin α11β1), coupled with dose-limited, or locally delivered CXCR4 inhibitors, could help to temporarily impair retention of the immune cells, movement of the FLS, osteoclastogenic signals, and support for the synovial microenvironment (95). Agents such as AMD3100/plerixafor, or newer CXCL12/CXCR4-regulating agents such as advanced EPI-X4 analogs, could thus serve as peri-infusion conditioning agents of the microenvironment. This will require meticulous regulation of the drug dose, timing, route of administration, and appropriate biomarkers to prevent prolonged interference with the normal trafficking of blood cells. It is crucial to note that CXCR4 inhibitors cannot be used concurrently with CAR constructs designed to overexpress CXCR4 for targeted homing to the joint tissues. In such cases, CXCR4 inhibition should either be avoided or temporally separated from the homing phase. This completes the design logic: CXCL12/CXCR4 is best positioned as a pathogenic amplifier and a combination target, rather than a safe antigen for direct CAR-mediated depletion (96, 97).

#### 3.2.7 Other ECM−associated targets

Numerous extracellular matrix (ECM)- and stroma-associated proteins have now been validated as secondary targets to cell-surface FLS antigens, enabling disruption of the pathogenic synovial niche rather than targeting a single cellular lineage. An example from this class is CD248 (endosialin), a transmembrane glycoprotein that is induced on activated FLS and perivascular stromal cells in RA but is minimally expressed in healthy synovium (98). Genetic ablation of CD248 greatly reduces synovial hyperplasia and inflammation in preclinical models, suggesting its functional relevance (99). Tissue-destructive programs are also engaged when ECM is incited by integrin α11β1, a collagen receptor upregulated on RA-associated fibroblasts, and its relatively limited stromal distribution makes potential binding sites attractive (90). In contrast, widely expressed ECM constituents or enzymes like specific fibronectin splice domains, collagens (as read out via integrins), heparanase, tenascin-C, and collagen VI are largely extracellular and thus less well suited to direct cell-targeted strategies, but may respond to local delivery approaches such as matrix-directed antibodies/enzyme inhibitors/ADC (100, 101). (**Table 1**).

**Table 1:** Candidate FLS surface antigens for targeted engineered therapies in rheumatoid arthritis.

Antigen	Role in RA pathogenesis	Main advantages	Key safety / limitation concerns	Best-fit engineering strategies	Ref.
CDH11	Maintains synovial lining architecture, Supports FLS adhesion, Pannus persistence, and IL-6 production	Strong link to synovial fibroblast biology, More FLS-focused than broad mesenchymal markers	Not RA-specific and low-level expression in other mesenchymal tissues	Affinity-tuned CAR-T/NK, Local delivery, AND-gated or adaptor CAR systems	(73, 102)
FAP	Synovitis, Matrix remodelling, Cartilage damage, Bone erosion	Strong disease-associated stromal marker, Supported by imaging and functional depletion studies	Expressed during wound healing and fibrosis, Systemic targeting may affect non-synovial repair tissues	Local CAR delivery, transient mRNA CAR, BiTE/adaptor systems, logic gating, hypoxia-gated designs	(103)
PDPN	Promotes FLS migration, Cartilage invasion, Osteoclast-supportive inflammation	Surface-accessible and disease-upregulated, Linked to invasive and flare-associated fibroblast phenotypes	Physiological expression in lymphatics, podocytes, and alveolar cells, Requires local or conditional targeting	Dual-antigen CARs, local delivery, Adaptor-controlled systems, Transient CAR-NK/mRNA CAR	(59, 104)
CD90 / THY1	Identifies IL-6/CXCL12-producing inflammatory fibroblasts	Useful for subset classification and patient stratification, Suitable as a combinatorial gating marker	Broad expression across fibroblasts and non-FLS lineages	Second input for AND gates, SynNotch circuits, or profiling-based target selection	(105, 106)
uPAR / CD87	Drives FLS invasion and migration, Matrix degradation, Angiogenic signalling	Relevant to pannus expansion and remodelling	Limited cellular specificity, Soluble uPAR and broad expression complicate direct targeting	Local or transient targeting, Pathway modulation, Adaptor-based approaches	(88, 107, 108)
CXCR4	Supports FLS migration, Leukocyte retention, Stromal–immune inflammatory crosstalk	Central RA chemokine axis, Useful for homing or microenvironmental modulation	Broad role in hematopoiesis and immune trafficking, Better suited for modulation than depletion	Chemokine receptor engineering, Local blockade, or Dose-limited CXCR4 antagonism	(109, 110)

### 3.3 Target validation strategies

**Figure 3:**
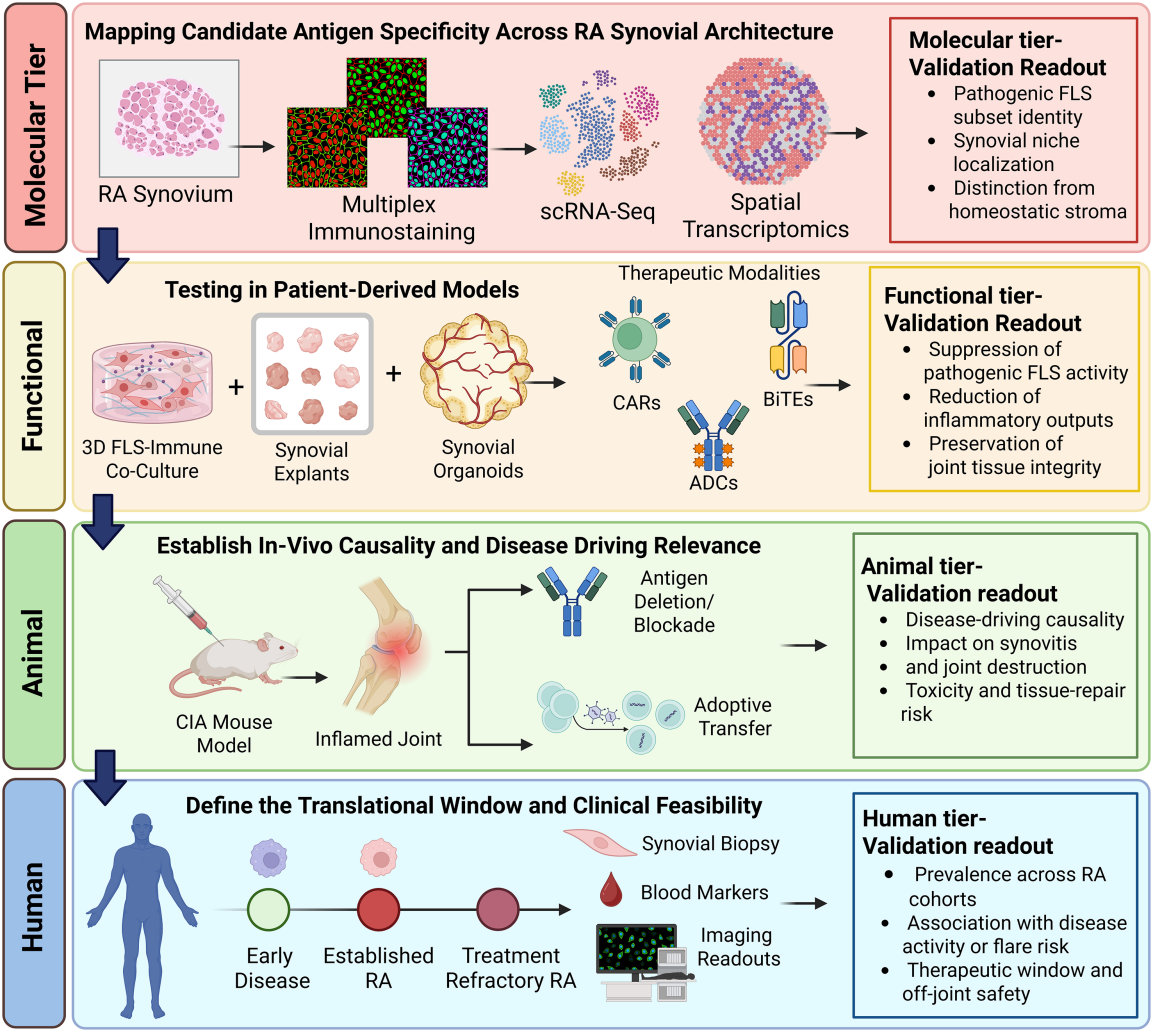
Tiered validation framework for FLS-directed antigen selection in rheumatoid arthritis. The schematic illustrates a stepwise validation strategy for selecting candidate antigens for FLS-directed therapies. Molecular profiling is first used to determine the localization of antigen expression in the RA synovium, particularly whether it correlates with pathogenic FLS subpopulations and niche locations. Functional validation through patient-derived co-cultures, synovial explants, and organoids will verify whether targeting the antigen reduces inflammation and tissue destruction in FLSs without damaging the joint. The animal study will further validate the role of the antigen in the disease pathogenesis. Human validation finally confirms disease-stage relevance, cohort prevalence, biomarker or imaging association, and off-joint safety to define the translational window for CAR-, BiTE-, ADC-, or related FLS-directed approaches.

For FLS-directed therapy, antigen selection should not be based solely on expression enrichment. The antigen must meet a validation process that proves its molecular selectivity, functional significance in humans, causality *in vivo*, and translatability in clinical practice (**Figure 3**) (111). At the molecular tier, the selected antigen needs to be localized throughout the rheumatoid synovial architecture using single-cell RNA sequencing, spatial transcriptomics, CITE-seq or CyTOF, and multiplex staining (26, 28, 112). The rationale is to demonstrate the antigen’s presence in pathogenic fibroblast phenotypes, not in normal stromal cells. The functional validation should proceed to patient-derived platforms that mimic the cellular and microenvironmental complexity of the diseased joint. Human synovial explants, 3D co-cultures of FLS and immune cells, and synovium organoids must be used to assess whether the engagement of targets by antibodies, CAR-modified cells, BiTEs, and ADCs blocks key pathogenic functions, including IL-6, CCL2, CXCL12, MMP3, ADAMTS enzymes, RANKL, fibroblast invasion, and matrix remodeling. At the same time, it must be validated that chondrocytes, endothelial cells, macrophage homeostasis, and normal stroma are intact (113–115).

Animal validation should establish whether the target is merely a marker of activation or a true disease-driving node. This requires antigen deletion, blockade, depletion, or adoptive-transfer approaches in relevant arthritis models, with clear assessment of synovitis, cartilage damage, bone erosion, systemic toxicity, and tissue repair (116). Strong precedents already exist: FAPα-positive fibroblast subsets separate inflammatory and tissue-destructive functions, FAP deficiency reduces cartilage destruction in inflammatory arthritis, and transfer of ITGA5-positive synovial fibroblasts aggravates collagen-induced arthritis, directly linking defined fibroblast states with disease amplification (66, 117, 118). Finally, human validation should confirm that the antigen is prevalent, disease-stage relevant, and therapeutically safe across independent RA cohorts. This requires testing synovial biopsies from different disease stages and treatment backgrounds, blood-based biomarkers, imaging readouts, and non-joint tissue panels to define the true therapeutic window (119, 120). The rise of PDPN-positive PRIME cells before clinical flare and the ability of FAP-based imaging to localize activated stromal tissue in RA patients demonstrate how human validation can link fibroblast biology to disease activity and clinical feasibility (78). Therefore, only targets supported by molecular specificity, functional suppression in human tissue, *in vivo* causality, and patient-level safety should advance toward CAR, BiTE, ADC, or related FLS-directed therapeutic development.

## 4 Engineering immune cells to target FLS: platforms and design options

### 4.1 CAR-engineered immune T cells: design basics and lessons from oncology

Immune-cell engineering has evolved from being an oncology innovation to a broadly translatable therapeutic platform. CD19-targeted and other chimeric antigen receptor (CAR) T products result in high objective and complete response rates in B-cell malignancies, supporting the notion that genetically reprogrammed lymphocytes can mediate prolonged, disease-modifying remissions (121). These clinical successes have hastened attempts to repurpose engineered cellular therapies for autoimmune disease; early case series of systemic CD19 CAR-T therapy demonstrated deep, sustained remissions in refractory autoimmunity, providing proof-of-principle evidence for the application of an “immune-reset” strategy (11, 122). Using that paradigm, novel, payload-laden architectures (for example, autologous CAR-T cells engineered to co-express neutralizing single-chain fragments specific for IL-6 and TNF-α) have exhibited rapid, durable responses in treatment-refractory rheumatoid arthritis as a demonstration of how the combinatorial combination of cellular depletion with local cytokine modulation can rewire the inflammatory niche (123). Importantly, CAR T cells can target and ablate pathogenic synovial fibroblasts, but RA presents unique challenges.

In RA, CAR-T cells are likely to be most useful in patients with severe, invasive, and erosive synovitis, in which pathogenic FLS are markedly expanded and express activation-associated markers such as FAP and PDPN (51). Among the candidate FLS-directed targets, FAP is especially attractive for CAR-T design because it is enriched in activated stromal cells and has shown therapeutic potential in experimental fibrosis (124). However, because FAP can also be expressed during wound healing and tissue repair, unrestricted systemic FAP CAR-T therapy would carry a risk of off-tissue stromal injury. This means that an RA-specific FAP CAR-T strategy must consider additional safety measures, including affinity-based modification of antigen recognition, temporary CAR expression, hypoxia-based regulation, localized delivery, or dual gating with another antigen present on FLS. PDPN could prove especially important in cases of more aggressive pannus-like RA, given its presence in migrating and bone-resorbing FLS (59). Despite this, PDPN’s localization to lymphatics, podocytes, and alveolar epithelium reduces the feasibility of systemic treatment, making localized, temporary, or conditional treatment more feasible (77, 125). This is not the case with CD90, which cannot be used as the sole CAR-T target. In contrast, CD90 should not be considered a standalone CAR-T target due to its broad stromal and non-stromal distribution. Instead, CD90 may be more valuable as a secondary input in an AND-gated design, such as FAP/PDPN combined with CD90 or HLA-DR, to enrich CAR activation within inflammatory sublining FLS while sparing homeostatic fibroblast populations (28, 126).

The CAR structure also matters. The extracellular hinge length should match the antigen’s orientation (short CD8α hinges favor membrane-proximal epitopes, while longer IgG4 hinges can reach distal ones). For signaling, one must choose co-stimulation carefully (127, 128). In oncology, CARs using CD28 co-stimulators unleash rapid, potent cytotoxicity (with brisk cytokine release), whereas 4−1BB-containing CARs tend to drive more sustained memory and lower peak inflammation. For RA, it may be prudent to favor 4−1BB (or test both); a 4−1BB CAR might clear FLS steadily while minimizing cytokine surges (129). Third-generation or “armored” CARs (co-expressing cytokines or dominant-negative receptors) can boost activity in hostile environments, but each addition escalates risk (130). Given the novelty, first-in-human RA trials should start conservatively. One approach is to use transient CAR expression (via mRNA electroporation or lipid nanoparticles) so that cells naturally disappear within days. Indeed, transient mRNA CAR-T strategies are now beginning to move beyond oncology and into autoimmune diseases. A recent example is BCMA-directed mRNA CAR-T therapy in patients with Myasthenia Gravis, where exploratory biomarker findings from a placebo-controlled phase 2b trial provided early evidence that RNA-engineered cellular therapies can be applied safely and feasibly in non-malignant immune conditions (131). Efficacy should be assessed in humans using synovial explants or 3D RA organoids that one would anticipate would kill FLS and inhibit their IL-6/MMP secretion, without negatively impacting chondrocytes. Off-target screens (e.g., primary human binding arrays or cross-referencing single-cell atlases) are crucial for verifying the absence of off-tissue hits (111). *In vivo* models can be monitored for safety via imaging (MRI or PET) and serum biomarkers of joint damage (e.g., collagen CTX-II); cytokine panels from synovial fluid versus serum would discriminate local inflammation in the relevant joints from systemic CRS (132). Crucially, clinical protocols must lean toward placing locoregional therapies. As with intratumoral CAR-T trials, direct intra-articular injection of FLS−CAR-T (especially in a large joint such as the knee) should concentrate the effects and dramatically reduce systemic exposure. Indeed, a clinical study of intratumoral mRNA CAR-T cells reported only minor grade-1 toxicity despite robust local tumor killing (133). In RA, a similar strategy in a sentinel joint, with slowly escalating cell doses and planned suicide-switch activation procedures, would maximize safety while allowing direct readout of synovial response.

### 4.2 CAR-NK and iPSC-derived NK cells

Natural killer (NK) cells offer complementary advantages. They do not cause graft-versus-host disease, so healthy-donor (allogeneic) NKs can be used off-the-shelf (134). NKs possess innate cytotoxicity via perforin/granzyme, natural cytotoxicity receptors (NKp30/46, NKG2D), and antibody-dependent cellular cytotoxicity (via CD16) (135, 136). This means a CAR-NK can kill a target by both its CAR and native mechanisms, potentially enhancing clearance of antibody-opsonized fibroblasts or stressed cells. Because NKs have different receptor repertoires, they may recognize aspects of the fibrotic synovium that T cells miss (137). When engineering CAR-NKs, many design principles parallel those for CAR-Ts, with NK−specific tweaks. For example, NK-optimized signaling motifs such as DAP12 or 2B4 (CD244) can be used in place of or alongside CD3ζ (138). In fact, recent work showed that CAR constructs incorporating NK−derived costimulatory domains (such as 2B4 or DNAM-1) drove much higher persistence, proliferation, and cytotoxicity in NK cells than the conventional T-cell CD28 domain (139). Likewise, using transmembrane and adaptor elements from NK receptors (e.g., the NKG2D or NKp46 transmembrane region) has been shown to enhance NK-CAR activity. CAR-NK cell protocols also commonly include an IL-15 signal (140). This can be in the form of co-expressed membrane-bound IL-15, which provides autocrine survival signaling. For instance, the NKX101 CAR-NK uses a membrane IL-15 construct to extend *in vivo* persistence (141).

Additional evidence from iPSC-derived NK-cell platforms supports the ability to standardize and genetically engineer off-the-shelf cellular therapies. Studies in preclinical models have demonstrated that genetically modified iPSC-derived NK cells with high-affinity, non-cleavable CD16 can increase antibody-dependent cell-mediated cytotoxicity. At the same time, multiplex genetic modification approaches have added additional traits, including CAR specificity, membrane-bound IL-15 expression, and CD38 knockout (142). The development of FT596, an iPSC-derived CD19 CAR-NK cell therapy that carries a high-affinity, non-cleavable CD16-IL-15 receptor fusion, provides further evidence for the feasibility of creating off-the-shelf NK cells clonally derived from iPSC master cell lines. Although the aforementioned studies were conducted primarily in oncology, they still provide evidence of the platform capabilities of iPSC-derived NK cells (143, 144).

This limited persistence may be particularly advantageous in RA, where several candidate targets are enriched in the inflamed synovium but still contribute to normal stromal structure and tissue homeostasis. CDH11 is a relevant example, as it plays an important role in synovial lining organization and FLS adhesion (145). In this context, a shorter-lived CAR-NK or iPSC-derived CAR-NK platform may provide a more controlled means of targeting activated CDH11-positive FLS while avoiding prolonged depletion of synovial structural cells that may still contribute to tissue organization (146). A similar argument can be made for collagen-binding integrin α11β1, which is involved in FLS adhesion, migration, and extracellular matrix interaction, rather than being a disease-exclusive antigen (90). For this reason, CAR-NK cells or locally delivered NK-based approaches may be safer and more practical for α11β1-directed therapy than durable systemic CAR-T cells. In mild-to-moderate polyarticular RA, repeated dosing with off-the-shelf CAR-NK cells against CDH11, selected integrins, or other lower-risk FLS-enriched targets may therefore be more clinically realistic than long-persisting systemic CAR-T therapy (145, 147).

### 4.3 Alternative effectors

Apart from CAR T/NK, several novel modalities could be used to target FLS. In principle, TCR-engineered T cells could target fibroblasts presenting intracellular autoantigens (such as the citrullinated peptides that are presented by shared-epitope MHC-II alleles), but such strategies face challenges from HLA restriction and potential TCR cross-reactivity; thus, any TCR-T strategy would need to carefully define exhaustively validated RA-specific peptide-MHC targets (e.g., PAD4 or citrullinated vimentin peptides in the relevant HLA context) to minimize the risk of catastrophic off-tissue recognition (148–151). Cell-free bispecific T-cell engagers (BiTEs) provide an alternative; a CD3–anti-FAP or CD3–anti-PDPN BiTE would intra-articularly recruit endogenous T cells to lyse proximal pathogenic FLS while providing easy dose titration or discontinuation, but systemic leakage risks unwanted peripheral activation of T cells and cytokine release, and the short half-life (ca. 1–2 hours) for most BiTEs may require continuous infusion or chemical half-life extension (152, 153). Notably, Bucci et al. (2024) reported compassionate-use treatment of six patients with multidrug-resistant RA using the CD19×CD3 BiTE blinatumomab; low-dose administration produced rapid clinical and imaging improvement, depletion of pathogenic B-cell subsets with an immune reset, and lowering of autoantibodies, thus supporting the ability for this therapy to achieve clinical feasibility in RA (154).

This clinical experience supports the broader idea that short-acting immune engagers may be useful in RA when the target is biologically relevant but not safe enough for durable systemic CAR-T. Accordingly, PDPN, uPAR, CD90, and CD24 may be better suited to locally delivered BiTEs, adaptor CARs, or other titratable systems than permanent CAR-T cells (81, 154). For example, uPAR is attractive in invasive or senescent-like FLS because it is linked to matrix remodeling and cell migration, and uPAR-directed CAR-T cells have shown senolytic activity in published fibrosis and aging-related models; however, because uPAR is also expressed on myeloid and endothelial compartments, RA applications should favor local, transient, or modular targeting rather than uncontrolled depletion (155, 156). CD24 should be viewed as an exploratory target in RA, but it may be most relevant in macrophage-linked or adaptor-based CAR strategies. This is because CD24 can act as a “do-not-eat-me” signal through Siglec-10, thereby limiting macrophage-mediated phagocytosis. Likewise, CD248 and ECM-associated targets may be better matched to CAR-macrophages, extracellular vesicles, or matrix-directed local delivery, as their main value lies in stromal remodeling, phagocytic clearance, and niche reprogramming rather than direct cytotoxic elimination alone (157, 158).

**Figure 4:**
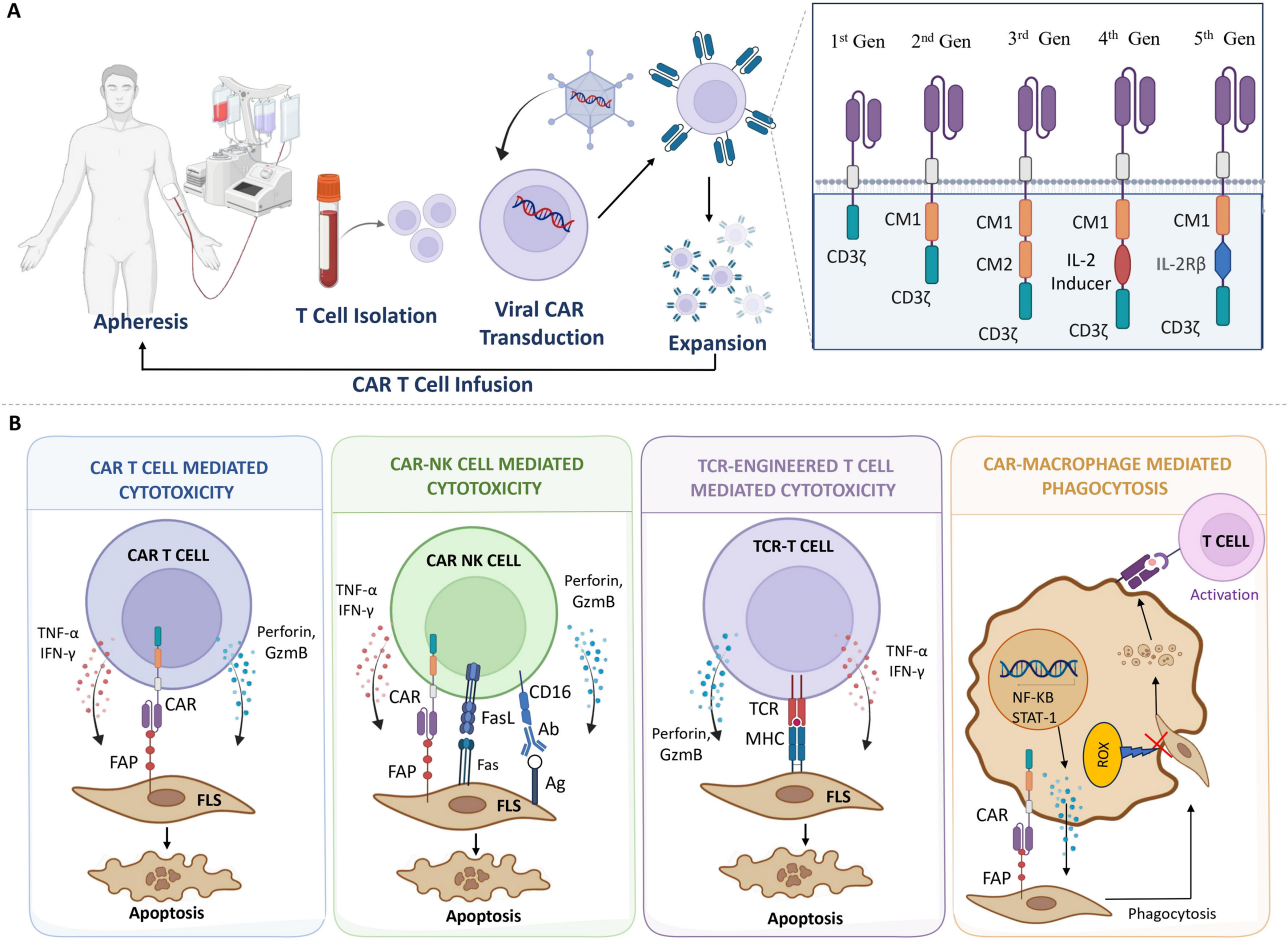
Engineered cell therapy platforms targeting fibroblast-like synoviocytes. **(A)** Overview of the CAR-T cell development process, starting with leukapheresis, followed by T-cell isolation, viral transduction, ex vivo expansion, and reinfusion into the patient. The inset illustrates the evolution of CAR designs from first- to fifth-generation constructs. **(B)** Comparison of engineered cell therapies targeting fibroblast-like synoviocytes, including CAR-T cells, CAR-NK cells, TCR-engineered T cells, and CAR-macrophages.

CAR-macrophages (CAR-M) merge both phagocytic clearance with stromal remodeling; therefore, engineered CAR-M targeting FLS and polarized towards a pro-resolution phenotype could remove fibrotic debris and produce anti-inflammatory mediators (159, 160). In RA, this platform is particularly relevant because the inflamed synovium is rich in macrophages and invasive FLS, and their reciprocal crosstalk drives the production of persistent IL-6, TNF-α, and GM-CSF, as well as MMPs, pannus formation, cartilage invasion, and osteoclast-mediated bone erosion. But the potential of CAR-M to secrete proteases poses a serious risk of unwanted cartilage or bone degradation unless stringent safeguards (inducible expression of degradative enzymes, suicide switches, or other off-switches) are incorporated (161). Engineered exosomes/extracellular vesicles are a non-cellular delivery platform for siRNA, miRNA, or protein deliveries (e.g., MMP7, NOTCH3, and miR-140 mimetics were discussed) that can tolerate surface decoration to enhance synovium targeting and stability (e.g., through ligands or PEGylation) (162). For RA, EV-based approaches may be especially useful for delivering cargo that silences FLS-intrinsic pathogenic programs, including NF-κB, STAT3, NOTCH3, MMPs, RANKL, or pro-invasive cytokine circuits, without introducing a long-lived engineered immune cell into chronically inflamed joints (163). Across these modalities, the preclinical threshold is the same. They demonstrate selective action on pathogenic FLS (decreased invasiveness, lower levels of IL-6/MMP secretion, and reversal of EMT-like markers) while maintaining chondrocyte, bone, and tendon cell viability and normal fibroblast function; only approaches that combine potent, joint-confined efficacy with limited bystander damage should progress into clinical translation (164) (**Figure 4**; **Table 2**).

**Table 2:** Comparative analysis of engineered cellular and non-cellular platforms for FLS-directed therapy in rheumatoid arthritis.

Platform	RA-specific rationale	Main advantages	Main limitations / barriers	Design / safety mitigations	Ref.
CAR-T cells	Targets pathogenic FLS driving pannus, cytokine release, matrix damage, and osteoclast activation, Best suited for severe or refractory synovitis	Highly potent and antigen-specific, Can be engineered with logic gates, safety switches, or armoring modules	Long persistence may be risky in chronic RA, Imperfect antigen specificity may cause stromal off-target toxicity	Affinity tuning, suicide switches, Transient mRNA CARs, local delivery, AND/NOT gates, adaptor control	(165, 166)
mRNA / transient CAR platform	Provides short-term FLS targeting without durable cell engraftment, Allows repeated dosing with safety reassessment	Reversible, titratable, and repeatable, Well suited for local and dose-controlled therapy	Short activity window, Repeated dosing may be needed, especially in dense synovium	Low-dose repeat schedules, local delivery, Biomaterial retention, imaging-guided redosing, background DMARD support	(167, 168)
CAR-NK / iPSC-NK cells	Offers FLS-directed killing with shorter persistence than CAR-T cells, Off-the-shelf platforms may suit chronic polyarticular RA	Lower theoretical GvHD risk, Scalable production with innate cytotoxicity	Limited persistence and expansion, Homing into fibrotic synovium may be inefficient	IL-15 supports, Chemokine-receptor engineering, Local delivery, Repeat dosing, optional safety switches	(169, 170)
CAR-macrophages / CAR-M	Relevant to macrophage-rich RA synovium, May disrupt macrophage–FLS crosstalk and clear inflammatory debris	Combines antigen recognition with phagocytosis Can be programmed toward pro-resolution functions	Macrophage phenotype is difficult to control Excess tissue remodelling may harm cartilage, tendon, or bone	Pro-resolution programming, Inducible modules, strict off-switches, local delivery, cartilage/bone safety testing	(171, 172)
BiTE / adaptor-controlled redirectors	Useful when FLS antigen specificity is incomplete, Enables temporary and dose-controlled immune redirection	Reversible, modular, and rapidly stoppable, Useful for testing new FLS targets	Short half-life may require repeated dosing, Systemic leakage may activate peripheral T cells	Local delivery, short-acting adaptors, Split dosing, Imaging-based monitoring	(154, 173)

## 5 Safety-first design principles

### 5.1 Antigen-level safety: affinity tuning and epitope selection

A fundamental strategy to improve specificity is to tailor the CAR’s antigen recognition to features unique to diseased FLS. This begins with affinity tuning. For example, Shabaneh et al. confirmed that higher-affinity anti-HER2 CARs invaded and eliminated normal HER2+ tissues, leading to lethal toxicity, whereas a lower-affinity HER2 CAR controlled tumors without damaging normal cells (174). Analogously, anti-FLS CARs should employ a scFv of intermediate-affinity: tight enough to be lethal for FLS (which express elevated amounts of the target antigen) but loose enough that normal cells, which only express low levels, could be ignored (175). For RA, the affinity threshold should therefore be calibrated against antigen density across active synovitis, remission-stage synovium, osteoarthritis synovium, and non-synovial fibroblast-rich tissues, since the same marker may be expressed at different levels across disease stage, joint site, and stromal compartment (176). Nominal scFvs would be checked for minimal binding at the physiological antigen density on panels of healthy human tissues. Epitope selection is also a key factor beyond affinity. If feasible, one should aim for disease-specific epitopes or isoforms. EGFRvIII, a deletion mutant of EGFR present exclusively in glioblastoma, is an example of this; CARs against EGFRvIII do not attack normal tissues expressing wild-type EGFR (177, 178).

In RA, analogous strategies might include targeting neo-epitopes generated by inflammation, such as citrullinated peptides (if displayed on FLS MHC) or cleavage-specific fragments (179). Podoplanin (PDPN) provides an RA-relevant example: it is upregulated on activated synovial fibroblasts but absent in healthy synovia, and anti-PDPN antibodies ameliorated arthritis in mouse models (60). If a PDPN-directed CAR is developed, its epitope should ideally be restricted to the form expressed only on inflamed FLS. A similar method can be applied to FAP, which is enriched on activated RA-FLS and has recently been exploited for FAPI-PET visualization of inflamed joints; however, because fibroblast activation can also occur in tissue repair and extra-articular fibrotic lesions, FAP-directed CARs would require careful affinity tuning or epitope restriction rather than broad high-affinity targeting (180). More generally, the CAR’s target should be screened for in diseased and healthy tissues at the biochemical level. Differential glycosylation or protease-processed forms of a surface protein can serve as safeguards (181, 182). An scFv that recognizes a specific glycoform or splice variant present on FLS but not on healthy fibroblasts would dramatically raise the bar for specificity. In sum, designing antigen recognition with moderate affinity and a focus on disease-restricted epitopes can create a therapeutic window. As Shabaneh et al. conclude, using a low-affinity CAR “can safely regress tumors” with normal antigen expression, which is a lesson directly applicable to shared fibroblast antigens in RA.

### 5.2 Logic gating strategies

Even with careful antigen choice, no single marker may perfectly distinguish pathogenic FLS from all healthy cells. This limitation is particularly important in RA because the therapeutic target is not a malignant clone, but a heterogeneous stromal compartment that also contains homeostatic and pro-resolving fibroblasts required for tissue repair, synovial lubrication, and restoration of joint homeostasis. Therefore, logic gating in RA should be designed to recognize a pathogenic fibroblast state (especially like the ITGA5+ fibroblast subset we discussed in the previous section) defined by both lineage markers and inflammatory activation markers (118, 183). Logic-gated receptors can provide additional safety. “AND” gates require two antigens to trigger cytotoxicity. A prime example is the synNotch-CAR system: recognition of antigen A by a synNotch receptor induces expression of a CAR against antigen B, so only cells bearing both markers are killed (184, 185). In one study, a two-stage synNotch circuit required EGFR and MET (via two synNotch) to induce an anti-HER2 CAR; only cells co-expressing all three antigens were lysed (186, 187). We could imagine an RA-specific AND gate, such as “IF FAP and PDPN present, THEN activate CAR,” ensuring that isolated expression of either marker on normal cells is insufficient for attack. Similarly, parallel configurations exist where two separate synNotch receptors split a CAR into parts, only assembling a functional receptor when both are engaged. Multi-input AND circuits (3 or more antigens) have also been engineered to maximize precision. However, one must balance specificity against the risk of tumor/target escape when not all markers are co-present (183, 188).

“NOT” gates (inhibitory CARs, iCARs) act oppositely: binding a designated antigen shuts off the T cell. Fedorov et al. pioneered PD-1- or CTLA-4–based iCARs that deliver an inhibitory signal upon encountering a “safe” antigen (189). In their system, if a T cell simultaneously carries an activating CAR for antigen X and an iCAR for antigen Y, engagement of Y will transiently prevent the T cell from killing, even if X is also present. This effectively spares cells that express Y (for example, a vital normal tissue antigen) while allowing the CAR-T to kill cells that only express X (190, 191). Importantly, the inhibitory effect is reversible: after the encounter, the T cell can function normally again. In RA, one could imagine an iCAR targeting a ubiquitously expressed fibroblast antigen (e.g., a collagen marker) so that any FLS that unexpectedly shares that epitope with healthy stroma would be spared. Adaptor/modular CARs offer another axis of control. These systems use a “universal” CAR (for instance, an anti-Fc or anti-biotin CAR) plus a separate bispecific adaptor molecule that binds the target antigen and the CAR tag. Therapeutically, one can dose the adaptor to initiate or block activity, as Nixdorf et al. have successfully demonstrated in the treatment of AML (192, 193). If toxicity emerges, the adaptor can be withdrawn or competitively inhibited, immediately halting CAR engagement. This modular format also permits multiplexing; different adaptors can redirect the same CAR-T cells to different antigens and add an ON/OFF switch that resides outside the T cell itself (194). In an RA context, an adapter CAR could use an intra-articular infusion of an anti-FLS-antigen adaptor to initiate local activity, with the flexibility to halt targeting rapidly by stopping the infusion.

### 5.3 Temporal control

Safety can, in turn, be improved with tunable temporal gates, ranging from irreversible suicide genes for emergency ablation to reversible small-molecule brakes that enable real-time, dose-dependent modulation (195). In RA, temporal control has a distinct rationale because the therapeutic goal is not complete eradication of all FLS clones, but controlled suppression of pathogenic synovial activity while preserving normal stromal repair functions. The inducible caspase-9 (iCasp9) system, in which engineered T cells selectively express a synthetic caspase-9 fused to a drug-binding domain that is eliminated following administration of the chemical dimerizer AP1903, has been used in clinical trials to control CAR-T cells that lead to graft-versus-host disease (196). HSV-thymidine kinase (HSV-TK) is another irreversible suicide switch: it converts ganciclovir into toxic metabolites, thereby killing the engineered cells, and has also been explored in GD2-directed cellular therapy models (197). Because both approaches permanently ablate the therapeutic population, they are best reserved for emergency use. Reversible approaches permit temporary and titratable inhibition: in destabilizing-domain systems, Shield-1 stabilizes the CAR, whereas drug withdrawal leads to proteasomal degradation and loss of CAR expression (198). Split-CAR architectures use chemically induced heterodimerization, such as rapamycin-mediated FKBP–FRB pairing, to assemble signaling modules only in the presence of the drug (199, 200). Dasatinib and related agents provide an orthogonal means of transiently suppressing CAR signaling through kinase inhibition (201). Such reversible “pause” systems may be especially suitable for RA because they could allow temporary interruption of CAR activity during remission, infection, surgery, or excessive inflammatory responses, followed by reactivation when disease activity returns. This is relevant because RA-FLS can retain inflammatory and tissue-invasive programs even outside the acute inflammatory phase, making flexible rather than permanent control more appropriate for long-term disease management. Collectively, these systems offer real-time control over engineered-cell activity in line with patient status.

### 5.4 Spatial control

Targeting the anatomical footprint of engineered therapies greatly enhances safety by restricting activity in diseased tissue and minimizing systemic exposure. In RA therapy, the simple approach of localized administration (such as administering CAR-T or CAR-NK cells, or mRNA encoding CAR molecules, into a single affected synovium) can be used to build up localized concentrations of effectors while affecting only that one joint (202). An RA-specific frontier within this local-delivery concept is the use of injectable hydrogels, thermogelling materials, nanogels, and/or depot-forming biomaterials to extend the retention of CAR-encoded mRNA, adapter molecules, exosomes, or even short-lived engineered cells within the inflammatory synovial space (203, 204). In comparison to free injection into the joint space, such joint-localized systems would be able to offer controlled release, enhanced residence time in the synovium, and minimized systemic leakage, which becomes especially important due to the presence of chronic synovitis, joint effusion, increased vascular permeability, and fast biological clearance in RA joints. However, great care should be taken in developing their composition, degradation rate, injectability, immunogenicity, and effect on cartilage lubrication (205).

Although joint-localized delivery can substantially improve spatial safety, this strategy must be adapted to the clinical reality that RA is usually systemic, symmetrical, and polyarticular rather than confined to a single joint (206). Therefore, local administration should not be viewed solely as a single-joint intervention but as part of a broader spatial control strategy. A clinically realistic approach may be a “sentinel joint” strategy, in which one highly inflamed and accessible joint is selected for local delivery of CAR cells or adaptors. At the same time, systemic and imaging-based readouts are used to determine whether local disease suppression is accompanied by broader disease modulation (207). Notably, recent studies show that whole-body PET imaging with FAP-targeted tracers (^68Ga-FAPI) can quantify total synovial burden. In a 2023 RA trial, ^68Ga-FAPI PET/CT detected uptake in all known inflamed joints, and the PET-derived joint count correlated strongly with CRP and clinical disease activity. Such imaging (or analogous modalities such as MRI or ultrasound) provides a quantitative “whole-body” readout: the persistence or loss of CAR effects in the injected joint can be correlated with changes in remote joints on imaging (208). Moreover, intra-articular CAR-engineered immune cells could be administered alongside low-dose or background DMARD therapy rather than being used as a standalone intervention. This combined local–systemic approach has clinical precedent in RA treat-to-target strategies, such as the TICORA trial, in which intensive disease control involved escalation of systemic DMARDs together with intra-articular triamcinolone injections into actively inflamed joints, resulting in higher remission rates than routine care (209, 210).

Beyond combination with systemic therapy, chemokine receptor engineering offers another strategy to address the limitation of single-joint delivery. The RA-specific chemokine axis includes CXCL12–CXCR4, CCL2–CCR2, CCL5–CCR5, CXCL9/CXCL10–CXCR3, and CXCL13–CXCR5, mediating the recruitment of monocytes, T-cells, and B-cells into the chronically inflamed synovial membrane (211, 212). In an RA-specific application, a CAR against the FLS, when introduced into a sentinel joint, could overexpress CXCR4, CCR2, CCR5, CXCR3, or CXCR5, causing immune cells to migrate towards another joint with a matching chemokine gradient preferentially. The following findings in oncology have scientifically demonstrated this: CCR2b receptor-modified CAR-T cells have shown improved migration towards cancers secreting CCL2; CCR4 CAR-T cells have shown improved migration towards Hodgkin lymphomas secreting CCL17/CCL2; and CXCR1/2 CAR-T cells have shown improved migration towards tumors expressing IL-8/CXCL8 (213–215). However, because enhanced trafficking may reduce the safety advantage of local containment, this approach should be paired with temporal or pharmacological control systems, such as transient CAR expression, adaptor-dependent activation, suicide switches, or drug-regulated circuits (216, 217).

### 5.5 Immune dampening & collateral protection

Even a perfectly targeted CAR-T attack can spark local inflammation. To protect the joint’s healthy tissues and dampen bystander damage, cells can be co-engineered with immunoregulatory elements. One approach is to equip CAR cells with immune checkpoint ligands or anti-inflammatory cytokines (218, 219). For example, Boardman et al. used CRISPR to knock IL-10 into the PD1 locus of human CAR Tregs; upon CAR engagement, these Tregs secreted IL-10 and showed markedly improved suppression of autoreactive immune cells (220). For RA, this type of local immunoregulatory design may be particularly valuable because pathogenic FLS do not act alone, but maintain inflammatory loops with macrophages and lymphocytes that sustain synovitis even after the initial immune trigger has declined. Analogously, one could imagine CAR-T or CAR-NK effectors that, after killing FLS, also upregulate PD-L1 or secrete IL-10 to locally blunt T-cell and macrophage activation (221). These built-in “brakes” could limit cytokine-induced collateral damage. Another conceptual strategy is to have CAR-T cells express decoy receptors or scavengers (222, 223). For instance, cells could be engineered to overexpress IL-6R or TNF-binding domains to soak up excess inflammatory cytokines in the synovium. Or they could carry soluble CTLA-4 (abatacept-like) to inhibit antigen-presenting cells (224). While speculative, such protective cargo would turn the therapeutic cells into a self-regulating anti-inflammatory drug-release system, guarding against a runaway immune reaction. The inhibitory CAR (iCAR) concept mentioned above also serves this goal: a CAR-T cell carrying a PD-1–based iCAR can be programmed not to attack in the presence of a second antigen (for example, an epitope abundant on normal synovial cells). In practice, the most recent data suggest PD-1/CTLA4-based iCARs provide transient rescue from off-target attacks (225). Combined with checkpoint-ligand or cytokine armoring, these measures collectively help build a buffer around the therapy’s effector functions.

### 5.6 Hypoxia-gated CAR-T strategy

The inflamed rheumatoid synovium is a metabolically hostile niche: synovial fluid from patients is both hypoxic and acidic, with lactate concentrations reaching ~10 mM, and this environment drives a HIF-1α–dependent metabolic reprogramming in fibroblast-like synoviocytes (FLS) and infiltrating myeloid cells (226, 227). This makes hypoxia gating particularly relevant to RA because low oxygen tension is not merely a passive feature of inflamed synovium, but part of a self-sustaining stromal–immune circuit that supports FLS activation, cytokine production, immune-cell recruitment, and progressive joint destruction. Also, RA FLS upregulates key glycolytic enzymes such as HK2 and PFKFB3, increases lactate export, and acquires a migratory, pro-inflammatory phenotype that amplifies local IL-6 production while suppressing macrophage motility (228). These pathophysiological features can be exploited to improve the safety and precision of engineered cellular therapies: oxygen-responsive promoters (HREs) or oxygen-sensing domains (ODDs) have been incorporated into “hypoxia-gated” CAR designs to restrict effector expression to low-O_2_ microenvironments (229). In the study by Zhu et al., HRE-driven CEA-CAR-T cells exhibited a less differentiated and less exhausted phenotype *in vitro*. They produced more sustained and potent antitumor responses upon reaching hypoxic tumor sites than conventional CAR-T cells (230). Importantly, the first human phase 1 trial of the hypoxia-regulated CAR PC13 (NCT05396300), which uses an HRE-CAR targeting CEA, showed manageable toxicity and notable efficacy, with disease control rates exceeding 80% and objective response rates of around 57% in patients with increased CEA expression (231). For RA, hypoxia-gated CARs would need careful calibration across different joints and disease stages, as FLS phenotypes can vary between small and large joints. This would help ensure activation during active synovitis while limiting effects on homeostatic fibroblasts during remission.

### 5.7 Reducing persistence risk

Finally, controlling how long the engineered cells stick around can mitigate long-term risks. One approach is to use inherently transient expressions. mRNA-electroporated CARs or CAR mRNA delivered by LNPs confer antigen-specific function for only a few days (232). This has already been done in autoimmune trials; for example, a recent study showed the efficacy of CD19 CAR-Ts in murine lupus using mRNA CAR-Ts, allowing finely titrated activity with minimal toxicity (233). The same approach is being trialed in patients with refractory myasthenia gravis and could be applied to RA (131). Transient expression means any unexpected toxicity will dissipate within a week, and multiple low-dose administrations can be given as needed, with gaps to assess the effect. Another way to shorten persistence is to use short-lived effector cells. NK cells, γδ T cells, and even some macrophage therapies have limited lifespans *in vivo* (234, 235). For instance, clinical CAR-NK products typically persist for weeks to a couple of months, much less time than CAR-Ts. An iPSC-derived “adaptive NK” engineered with IL-15 lasted longer than normal NKs, but still far shorter than long-lived memory T cells (236). In the RA setting, one could deliberately choose a cell type (or remove cytokine support) to ensure the engineered cells naturally wane. This avoids the need for an active kill switch in many cases. A related tactic is dose fractionation. Instead of a single massive infusion, one could administer several smaller doses of cells (or adaptors), allowing assessment and recovery in between (237). This way, if any adverse signal arises, the treatment can be held or modified before the next dose (238).

**Figure 5:**
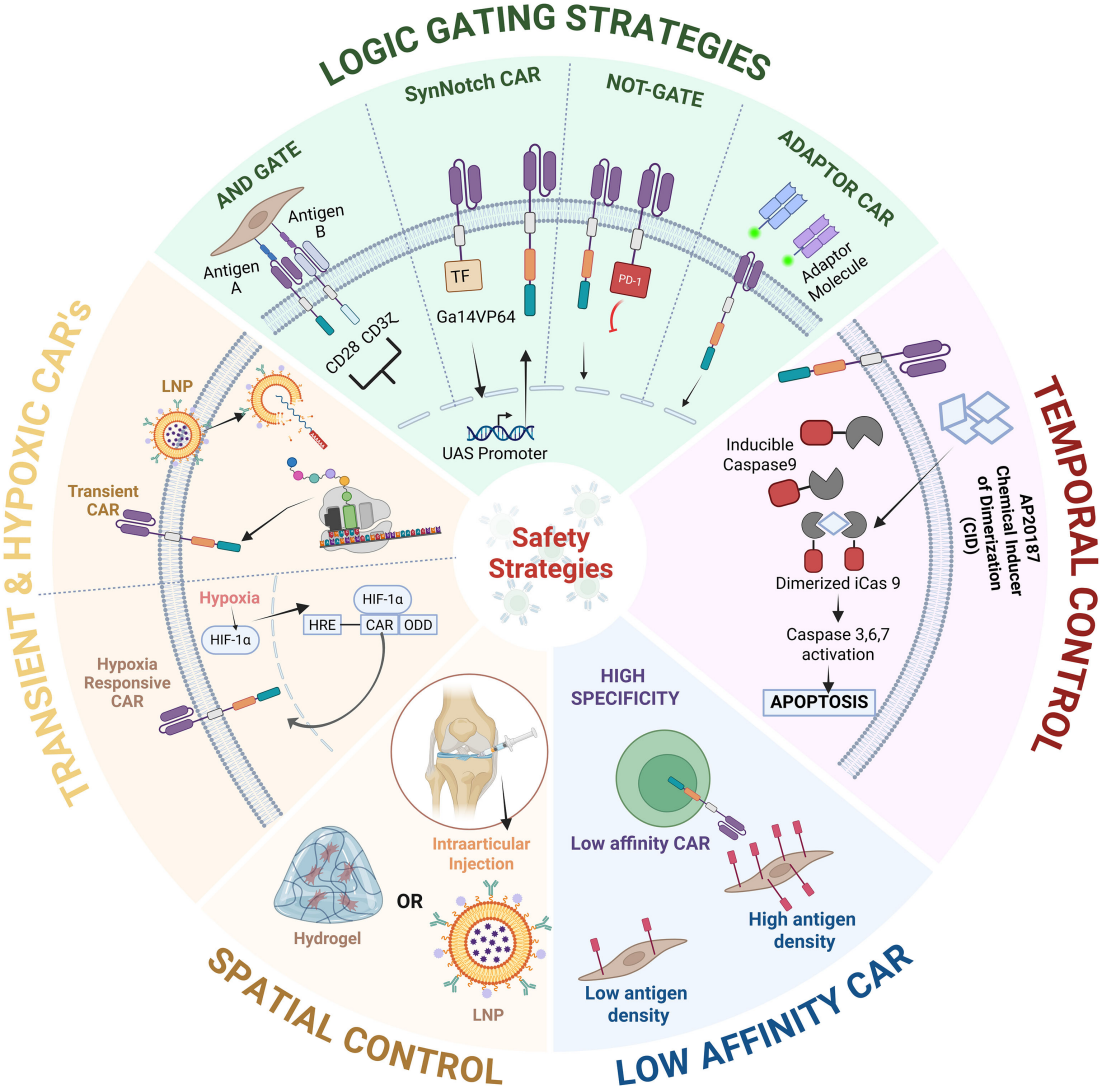
Multilayer safety strategies for CAR-T therapy in rheumatoid arthritis. The safety design strategies are organized into six complementary layers: adding logic-gating systems such as AND and NOT gates, synNotch circuits, and adaptor CARs; improving specificity with low-affinity CARs that preferentially recognize cells with high antigen density; using temporal control with inducible suicide switches and chemically regulated dimerization systems; limiting activity spatially through local delivery and biomaterial-based confinement; enabling transient and hypoxia-responsive expression with an mRNA CAR platform delivered via lipid nanoparticles, and with oxygen-sensitive elements such as HREs and ODDs; and reducing persistence to keep CAR activity short-lived *in vivo*.

On the other hand, repeated administration also presents the problem of immunogenicity. While transient expression of the CAR is safer, as it reduces the duration for which the modified T cells survive, repetitive exposure to the same CAR can result in its recognition and neutralization by the immune system (239). This possibility has already been demonstrated in early CAR studies: in one mRNA CAR-T study evaluating mesothelin-specific CAR-T cells, one of four subjects experienced sudden anaphylaxis and heart failure following the third infusion due to the production of IgE specific to the CAR (240–242). Likewise, in another CAIX-specific CAR-T study in patients with renal cell carcinoma, six of eleven participants developed human anti–CAIX-CAR antibodies (HACA) specific to the CAR idiotype, blocking its cytotoxic action *in vitro* (243). This is particularly crucial for RA therapy, which would require administering CAR fractions or transient CARs in multiple doses over time. To minimize such instances, future RA CAR constructs should preferably consist of either fully human or humanized antigen-binding sites and employ strategies such as deimmunization to avoid foreign epitopes (244). Previous evidence has shown that lymphodepletion with fludarabine/cyclophosphamide can minimize the anti-CAR antibody response, while repeat dosing remains possible. Short-course administration of corticosteroids, or even B-cell depletion therapy, could also play a key role if well balanced against the risk of infection (245, 246). Moreover, when murine or other antigen binders are used, pre-infusion testing for HACA or anti-mouse IgE would be prudent, especially for repeat administration in RA (**Figure 5**).

## 6 Limitations and future directions

Even the most promising FLS-targeted approaches are accompanied by inherent on-target, off-tissue risks. Because no single surface antigen is uniquely restricted to pathogenic FLS, some level of unintended collateral damage is difficult to avoid. For example, although fibroblast activation protein is highly expressed on RA-FLS, it is also found on normal stromal cells, and FAP-directed CAR T cells in murine models have been linked to severe bone toxicity and cachexia (68). In the context of rheumatoid arthritis, the therapeutic window is therefore likely narrower than in oncology. Systemic toxicity, particularly cytokine release syndrome or increased susceptibility to infection, remains the primary concern. Similarly, PDPN, uPAR, and CD24 may be biologically relevant in invasive, migratory, or macrophage-associated RA pathology, but their broader physiological expression patterns make unrestricted systemic targeting less feasible. These limitations support the need for safer designs (108, 247, 248).

A further challenge is that RA-FLS are highly heterogeneous and retain inflammatory memory even when removed from the inflamed joint milieu. Single-cell and spatial studies have shown that distinct FLS subsets contribute differently to cytokine production, matrix remodeling, cartilage invasion, osteoclast activation, and tissue destruction (249). Therefore, a single CAR target may either miss important pathogenic subsets or damage homeostatic fibroblasts. Inflammatory memory adds another barrier, because surviving FLS may remain epigenetically and metabolically programmed toward a pathogenic phenotype, allowing disease persistence or relapse after partial depletion (48). The identification of PRIME cells further complicates this picture, as circulating pre-inflammatory mesenchymal cells may expand before clinical flares and contribute to synovial inflammation, suggesting that RA stromal pathology is not always confined to established joint lesions. However, the fibrous nature of the collagen-rich extracellular matrix, vascularity, hypoxia, and polyarticular involvement in RA could impair the delivery and penetration of CAR cells into the tissue (250, 251). Collectively, these points illustrate that the primary translational hurdle is not only the selection of the antigen associated with RA but also the targeting of the individual patient’s stromal biology.

Because rheumatoid arthritis is chronic rather than self-limited, the ongoing generation of immune effector cells raises new safety concerns, including prolonged fibroblast depletion and unexpected autoimmune sequelae. These realities have made safety-first design principles a necessity (252). Future incarnations will likely incorporate combinatorial antigen gating, drilled down to AND/NOT circuits and synNotch-based logic, so that only synovium-specific antigen combinations trigger effector function. At the same time, similar cells elsewhere are spared (126, 253). Such built-in off-switches and fail-safe kill switches, like iCasp9, should be considered a must (254). The CAR itself can be engineered to be active in the synovial microenvironment, which is usually hypoxic and unique, for example, using hypoxia-responsive promoters or protease-cleavable masked CARs that prevent activity in healthy tissue. In the shorter term, transient or titratable platforms (e.g., mRNA-encoded CARs or drug-gated ON-switch CARs) may provide the most favorable efficacy-to-control ratio (255, 256). Equally important, patient selection based on multi-omic synovial profiling and emerging biomarkers of pathogenic FLS will help identify those most likely to benefit (257). Ultimately, the future of FLS-directed therapy will depend not on any single innovation, but on the integration of cellular logic, environmental sensing, pharmacologic control, and patient stratification into a coherent and clinically safe platform (258).

## 7 Conclusion

Although modern DMARDs have proved effective, rheumatoid arthritis cannot be fully controlled in many patients because current treatments do not target the synovial stromal network that sustains the disease. In this respect, immune cells engineered to target FLS represent a fundamentally different therapeutic approach: rather than simply dampening inflammation, they actively restructure the joint microenvironment. This is not ordinary cytotoxicity but precise engineering of the synovial ecosystem. Selective depletion of arthritogenic FLS or their reprogramming, which leaves healthy stromal cells intact, may push the tissue toward true resolution and away from chronic persistence. Realizing this vision will require safe, spatially constrained, and biologically specific designs. For now, the most feasible advances may arise from localized and tunable approaches such as intra-articular delivery, mRNA-based CAR expression, or ligand-inducible systems. Focused on the inflammatory FLS engine and with careful integration of synovial biology, synthetic engineering, and rigorous clinical testing, we believe that targeting the FLS burner could redefine how to achieve a cure for inflammatory arthritis.

## Glossary

RA: Rheumatoid Arthritis
FLS: Fibroblast-Like Synoviocytes
CAR: Chimeric Antigen Receptor
IL-6: Interleukin-6
TNF: Tumor Necrosis Factor
DMARDs: Disease-Modifying Antirheumatic Drugs
csDMARDs: Conventional Synthetic Disease-Modifying Antirheumatic Drugs
bDMARDs: Biologic Disease-Modifying Antirheumatic Drugs
tsDMARDs: Targeted Synthetic Disease-Modifying Antirheumatic Drugs
PDPN: Podoplanin
FAP: Fibroblast Activation Protein
CDH11: Cadherin-11
CAR-T: Chimeric Antigen Receptor T Cell
CAR-NK: Chimeric Antigen Receptor Natural Killer Cell
CAR-M: Chimeric Antigen Receptor Macrophage
ECM: Extracellular Matrix
CXCL12: C-X-C Motif Chemokine Ligand 12
CXCR4: C-X-C Motif Chemokine Receptor 4
MHC: Major Histocompatibility Complex
HLA: Human Leukocyte Antigen
JAK: Janus Kinase
LNP: Lipid Nanoparticle
HRE: Hypoxia-Responsive Element
iCasp9: Inducible Caspase-9
NK: Natural Killer
iPSC: Induced Pluripotent Stem Cell
scFv: Single-Chain Variable Fragment
BiTE: Bispecific T-Cell Engager
CITE-seq: Cellular Indexing Of Transcriptomes And Epitopes By Sequencing
synNotch: Synthetic Notch
TCR: T-Cell Receptor
PI3K/AKT/mTOR: Phosphoinositide 3-Kinase/AKT/Mechanistic Target Of Rapamycin
ADCC: Antibody-Dependent Cellular Cytotoxicity
ICANS: Immune effector cell-associated neurotoxicity syndrome
CRS: Cytokine release syndrome
